# Building qualitative research capacity in the global health workforce and developing a community of practice in parts of sub-Saharan Africa: a case study

**DOI:** 10.1186/s12909-026-09190-y

**Published:** 2026-04-24

**Authors:** Jack Lumsdon, Catherine Dotchin, Albert Akpalu, Benedict Calys-Tagoe, Dilraj Sokhi, Momodou Cham, Njideka Ulunma Okubadejo, Richard Morton, Violet Naanyu, Richard Walker, Matthew Breckons, Natasha Fothergill-Misbah

**Affiliations:** 1 Population Health Sciences Institute, Newcastle University, Newcastle upon Tyne, UK; 2 Translational and Clinical Research Institute, Faculty of Medical Sciences, Newcastle University, Newcastle upon Tyne, UK; 3 NIHR Newcastle Biomedical Research Centre, Newcastle upon Tyne Hospitals NHS Foundation Trust, Cumbria Northumberland Tyne and Wear NHS Foundation Trust and Faculty of Medical Sciences Newcastle University, Newcastle upon Tyne, UK; 4 University of Ghana Medical School, Korle Bu Teaching Hospital, Accra, Ghana; 5 Department of Community Health, University of Ghana Medical School, Korle Bu, Accra, Ghana; 6 Department of Neurology, Medical College, The Aga Khan University, Nairobi, Kenya; 7 Richard Novati Catholic Hospital, Sogakope, Ghana; 8 Neurology Unit, Department of Medicine, College of Medicine, University of Lagos, Lagos, Nigeria; 9 Northumbria Healthcare NHS Foundation Trust, Newcastle upon Tyne, UK; 10 School of Arts and Social Sciences, Moi University, Eldoret, Kenya

**Keywords:** Qualitative, Education, Global Health

## Abstract

**Background:**

Qualitative research offers unique insights into the lived experience of individuals with chronic conditions which can tie directly into public health priorities and identifying the need for additional support. More participatory approaches and qualitative data surrounding chronic diseases are needed in sub-Saharan Africa (SSA). Valuable “insider” insights are often missing as the conceptualisation of research often overlooks the value of such interactions. For successful and meaningful global health research, it is imperative to involve in-country experts and strengthen the capacity of research teams with qualitative skills.

**Methods:**

To address this, two hybrid qualitative research skills workshops were conducted in West Africa (Ghana; with virtual attendees from Nigeria) and East Africa (Kenya; with virtual attendees from Tanzania and Ethiopia) to deliver training and develop support networks as part of the ‘Transforming Parkinson’s Care in Africa’ research project. Both workshops were conducted over two days and involved presentations, discussions, and activities to ensure attendee interaction, while providing a comprehensive overview of qualitative research.

**Results:**

Materials were developed by facilitators, drawing on an experience of teaching qualitative methods and supporting research development in the UK, and doing qualitative research in SSA. Fifty-eight healthcare professionals and researchers attended the training. Following the training, short-term improvement was noted in self-rated knowledge and skills, with positive qualitative feedback from attendees. To ensure a lasting impact of the training, a community of practice has been established to provide continual support and mentorship to attendees. Five perpetual qualitative software licenses were also purchased for use by attendees and their teams to facilitate collaborative analysis of TraPCAf data and allow access beyond the duration of the project.

**Conclusions:**

There is a desire to develop qualitative research skills among researchers and healthcare professionals in SSA. This case study offers an example of how such training can be developed and delivered. To continue these learnings forward, a community of practice has been formed to ensure longer-term impact, with the ultimate goal of enhancing the qualitative research capacity of the public health workforce across SSA. This is enacted through continual engagement and mentorship of workshop attendees and addressing financial barriers to success by funding software access for individuals.

**Supplementary Information:**

The online version contains supplementary material available at 10.1186/s12909-026-09190-y.

## Background

Chronic diseases are one of the leading causes of disability and death worldwide with individuals experiencing poorer quality of life outcomes [1]. Chronic diseases account for around 50% of disease burden in low- and middle-income countries (LMICs) [2]. They contribute to increased costs for governments, communities, families, and individuals, and a loss of productivity for economies in Africa [2]. Qualitative research is vital to understand the nuances of experiences of chronic conditions to address the health, social and economic outcomes of individuals, and promote the economic growth of populations [3]. Qualitative research offers unique insight into understanding the lived experiences of individuals with chronic conditions to generate insights into the needs of these groups [4]. 

Understanding lived experiences ties directly into public health priorities and demands by identifying complexities in care pathways, medication (in)accessibility, and need for additional support [5–7].

Qualitative data surrounding chronic diseases is largely lacking across sub-Saharan Africa (SSA), with qualitative reviews consistently including less African literature than would be expected based upon prevalence [8–11]. Understanding the specific challenges people with chronic conditions, and their families, face places researchers and policymakers in a better position to identify and address setbacks in the patient experience across SSA [3]. In-depth understanding of these areas will primarily be informed by qualitative research, which will also guide campaigns to raising awareness and educate patients, their families, and healthcare professionals about public health priorities [12].

This work has been conducted in the context of the ‘Transforming Parkinson’s Care in Africa’ (TraPCAf) project; a multi-national multi-methods global health research group funded by the National Institute for Health and Care Research (NIHR) in the UK and hosted at Newcastle University [13]. The group includes researchers and clinicians from 7 African countries: Ethiopia, Egypt, Ghana, Kenya, Nigeria, South Africa, and Tanzania. Due to the varied nature of the study aims, researchers and clinicians have expertise across an array of methodological and clinical skills. One of these aims relates to understanding the experiences of people with Parkinson’s (PwP), their families, and healthcare professionals through the Qualitative Work Package. The data collection from seven African countries using qualitative methods provides a unique opportunity to gain insights across multiple cultures and health systems. The multidisciplinary and experienced research team is also well-positioned to provide both “insider” and “outsider” perspectives [13]. This is an essential component to put other findings of the research into perspective and gain understanding of what it is like to live with Parkinson’s, or care for PwP, in this context.

Through a survey across the seven TraPCAf, a needs analysis of research expertise identified qualitative research methodology as a significant gap across all sites. TraPCAf researchers identified minimal previous training and involvement in qualitative research as a knowledge and competency gap. For those with previous experience, involvement was often limited to data collection, with negligible opportunities to contribute to the analysis and dissemination of findings. This reflects the lack of prioritisation of lived experience and community engagement in the conceptualisation of research when developing solutions and policies within SSA [14]. Additionally, the inadequate number of qualitative researchers acts as a major barrier for teams when conducting qualitative research. Consequently, many funders and researchers are yet to be convinced of the benefits of qualitative research which further hinders the uptake of research exploring lived experience.

Researchers bring their own perspective and insights to qualitative data analysis, hence, not including African researchers within the analysis runs the risk of losing valuable “insider” insights into the data from people who work closely with the communities [15]. Across Africa, continuing professional development of individuals is often halted for a number of reasons including competing interests and priorities, lack of funding, and inadequate numbers of experienced trainers [16]. Thinking specifically about the public health research workforce across Africa, limitations in dedicated research teams and skilled research staff leads to barriers in conducting research [17]. Only by providing researchers in Africa with the opportunity to contribute, design, conduct, and create knowledge will we be able to produce meaningful research that reflects the experiences of populations under-represented in research [17, 18]. For successful and meaningful global health research, it is imperative to involve in-country experts working in Africa – and indeed other LMICs in other world regions which may have similar challenges – and strengthen the capacity of research teams with qualitative skills. This will result in future studies utilising robust qualitative methods and a better understanding of the intricacies and complexities of health [19, 20].

The TraPCAf team aims to establish a qualitative community of practice; a space whereby learning and information is shared between beginners and experts to promote professional and personal growth [21]. In this context, this is a virtual space in which mentorship is provided, good practice is shared, and challenges are discussed on an ongoing basis. Our goal is to build a cohort of researchers with skills to carry out novel and independent global health research to contribute to the scarce chronic disease literature from SSA using qualitative methods [8–11]. These data will ultimately address the health, social and economic outcomes of individuals, and contribute to appropriate interventions for the management of chronic disease across health and social care systems [3]. We anticipate these studies will contribute to more appropriate interventions for the management of chronic disease across health and social care systems, benefit underserved groups, improve the wellbeing of patients, and promote economic growth. This aim of this paper is to highlight this initial process of developing a qualitative community of practice, which will highlight the process of developing and delivering hybrid qualitative training, the short-term impact of the training, and the initial follow up to establish a qualitative community of practice.

## Methods

### Needs analysis

**Fig. 1:**
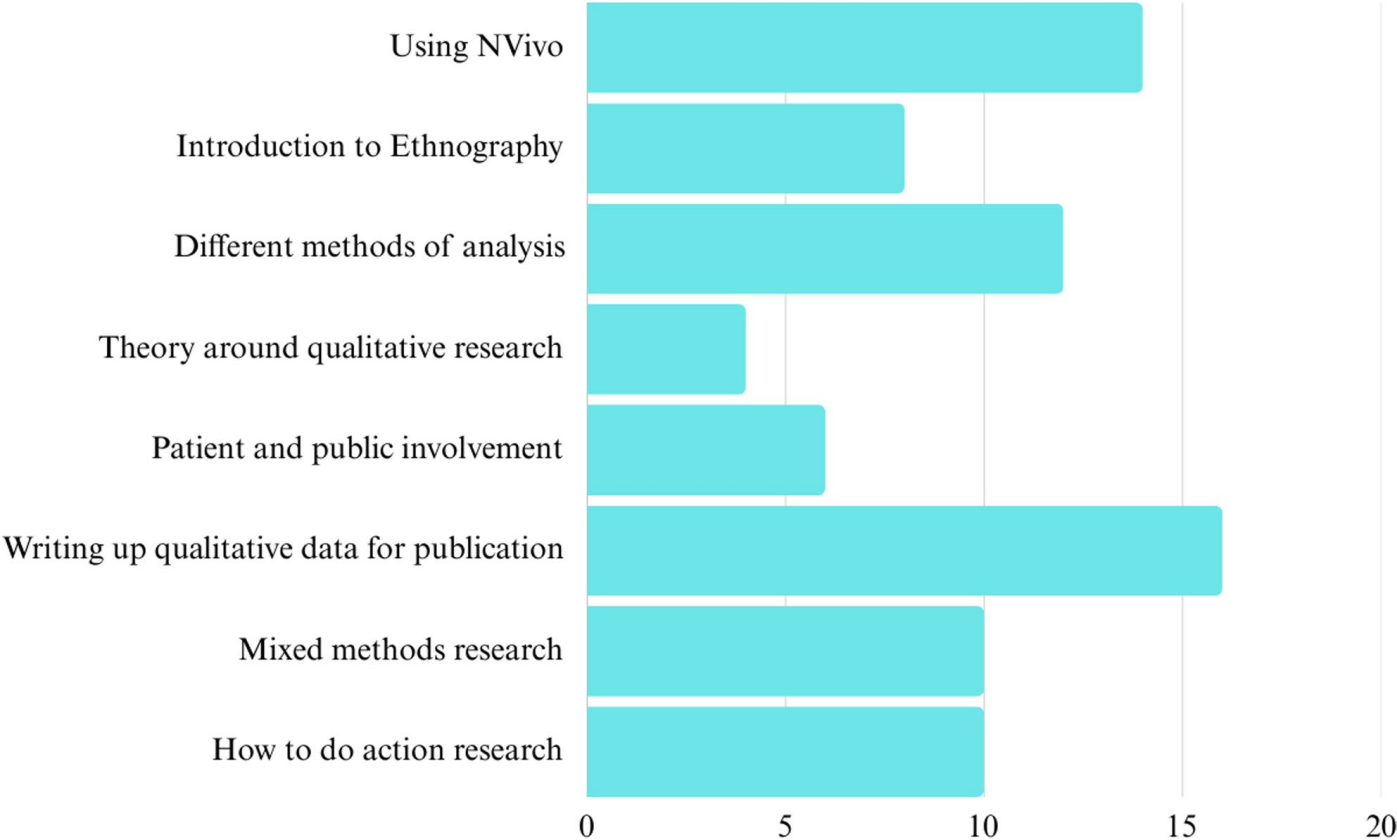
Training needs analysis following a qualitative research virtual introduction delivered by the TraPCAf team (*n* = 23)

Qualitative research, specifically qualitative health research in Africa, is an area of expertise at Newcastle University (NU), UK. The TraPCAf NU team hosted an initial virtual introduction to qualitative methods in early 2024 followed by an introduction to analysis a few months later. Forty researchers attended these sessions, including early career researchers (ECRs) and more senior co-investigators. 23/40 attendees completed a training needs survey which highlighted the demand for further in-depth training in ‘doing’ qualitative research, particularly around writing up qualitative data, use of NVivo^®^, and different methods of analysis (Fig. 1).

Funding was secured by the TraPCAf team through the NIHR via a Global Health Research Cohort Academic Development Award (CADA) to plan and conduct hybrid qualitative research training across the TraPCAf African sites.

### Training material development

The underpinning rationale behind the workshop content was to provide a practical ‘hands-on’ course, with a focus on developing the capacity of the TraPCAf qualitative research team. In our (NFM & MB) teaching experience, whether with undergraduates or post-graduates, there often appears to be a leap between learning about qualitative methodology, and its application. Students are often apprehensive about ‘getting started’, which we sought to focus on. This can be particularly challenging to overcome within a global collaboration in which co-researchers have limited experience in qualitative methods but have opportunity to contribute to data collection and analysis phases.

All workshop participants had attended online training sessions as part of the TraPCAf project, and the content of this workshop sought to build on this. The workshop provided a small amount of theoretical context and focussed on the practical elements of data collection and analysis, as these are more challenging to cover through online, presentation-based approaches. Materials were developed by facilitators, drawing on experience teaching research methods to a range of audiences including students at UK higher educational institutions and healthcare professionals, provision of methodological support through a research support service, and expertise in conducting qualitative research in SSA.

### Intervention delivery

Two hybrid qualitative research skills workshops were conducted over two days in West Africa (Ghana, with virtual attendees from Nigeria) and East Africa (Kenya, with virtual attendees from Tanzania and Ethiopia). Two qualitative researchers (NFM & MB) from Newcastle University travelled to Accra, Ghana and Nairobi, Kenya to present, chair, and facilitate the workshops. A qualitative researcher (JL) from Newcastle University facilitated virtual attendees via Zoom while based in the UK. Further, in-country facilitators (AA, MC, BCT, VN, RM, DS) from both within and beyond TraPCAf were present to ensure local representation and provide additional, contextually relevant, experience to draw from. The two workshops were held in September and October 2024.

The objective was to deliver training and develop support networks in each area. The workshops were recorded, and recordings were then disseminated to TraPCAf members across the 7 countries. A hybrid approach was chosen to balance the high costs of transporting all attendees to a venue, while ensuring as many attendees as possible benefited from the learnings. Despite trends towards virtual training sessions, largely due to the benefits this serves in terms of widening access, we felt purely virtual workshops would be less engaging and more difficult to coordinate. Hence, hybrid training was decided upon as the best approach, considering available funds. These workshops aimed to build and develop a cohort of researchers across the TraPCAf countries with skills in qualitative methodologies able to conduct independent studies. Invitations were made with a view of maximising value to country teams and the TraPCAf project, while extending invitations to a wider network of researchers and healthcare professionals where space allowed. Invitations specifically targeted ECRs who were anticipated to be collecting and analysing data, alongside more experienced researchers that would be able to supervise and support ECRs locally.

A training needs analysis and previous experience of facilitators delivering qualitative training at NU formed the basis of the content covered within the training workshops. A questionnaire was then sent to attendees prior to the workshop to understand perceived knowledge of topics and confidence in applying this to research, to further tailor the workshops to attendees’ needs. The workshops broadly involved content on qualitative theory and methodology, data collection and analysis, understanding of public and patient involvement and engagement, use of software to support analysis, and how to disseminate qualitative research (Supplementary Materials 1). To emphasise the value of rigor within qualitative research, content drew heavily on Rapley’s (2011) work to provide a pragmatic account of data analysis, with an emphasis on commonalities between research methodologies [22].

Both workshops were conducted in two days and involved presentations, discussions, and activities to ensure they were interactive, while providing a comprehensive overview of qualitative research. This was further facilitated online using breakout rooms and audiovisual technology in-person to allow those online to join the discussions. We acknowledge two days is not sufficient to encapsulate the entirety of the knowledge and skills required to conduct high quality qualitative research, hence we focussed on priority areas, providing the foundation for additional independent driven learning. Through discussions and engaging practical activities based on ‘real’ data, the attendees gained familiarity with the first-hand experiences of senior qualitative researchers as well as the ability to problem solve and apply the learnings to worked examples.

### Participants

The first workshop in West Africa had 27 attendees with in-country facilitators (AA, MC, BCT) and the second in East Africa had 31 attendees with in-country facilitators (VN, RM, DS) for a total of 58 attendees across both workshops, see Table 1. Sixty-four percent of attendees were considered early career. Ninety percent were researchers and 34% were healthcare professionals.

**Table 1:** Number of attendees for each workshop

Workshop	Country	Form of Attendance		Job Role			Career Stage		Total Attendees
		In-Person Attendees	Virtual Attendees	Researcher	Healthcare Professional	Both	Early	Mid and Late Stage	
West Africa	Ghana	19	1	15	3	2	14	6	20
	Nigeria	0	7	1	0	6	6	1	7
	Total	19	8	16	3	8	20	7	27
East Africa	Kenya	19	1	14	3	3	12	8	20
	Tanzania	1	5	3	0	3	3	3	6
	Ethiopia	0	5	2	0	3	2	3	5
	Total	20	11	19	3	9	17	14	31
Total	Total	39	19	35	6	17	37	21	58

### Data collection and analysis

This study employed a mixed-methods program evaluation design, combining pre-post quantitative surveys and qualitative feedback to assess the immediate impact of the training intervention. The anonymity of all participants was guaranteed, and the workshop posed no risk to the participants. Participation in the workshops was not dependent upon completion of pre- and post-questionnaires, each participant gave informed consent to participate in the workshop and data collection. The training questionnaire (Supplementary Materials 2) was developed to assess qualitative research knowledge and feedback on the workshop, this was circulated to attendees to evaluate the impact of the training. Sixty and 31 attendees completed the pre- and post-training questionnaires, respectively. As data from the questionnaires could not be matched between pre- and post-training questionnaires and was not normally distributed, a Mann Whitney U test was conducted in SPSS to compare the difference in self-rated knowledge and skills pre- and post-training. Open text responses were collected which were analysed through a deductive content analysis.

Eight of the qualitative workshop facilitators were invited to reflect on their experiences and involvement six months post-delivery. The facilitators were prompted to write a 300-word reflection on the qualitative workshops and the continued development of the qualitative skills of attendees. This was then analysed using a deductive content analysis.

## Results

### Participant experiences

**Fig. 2:**
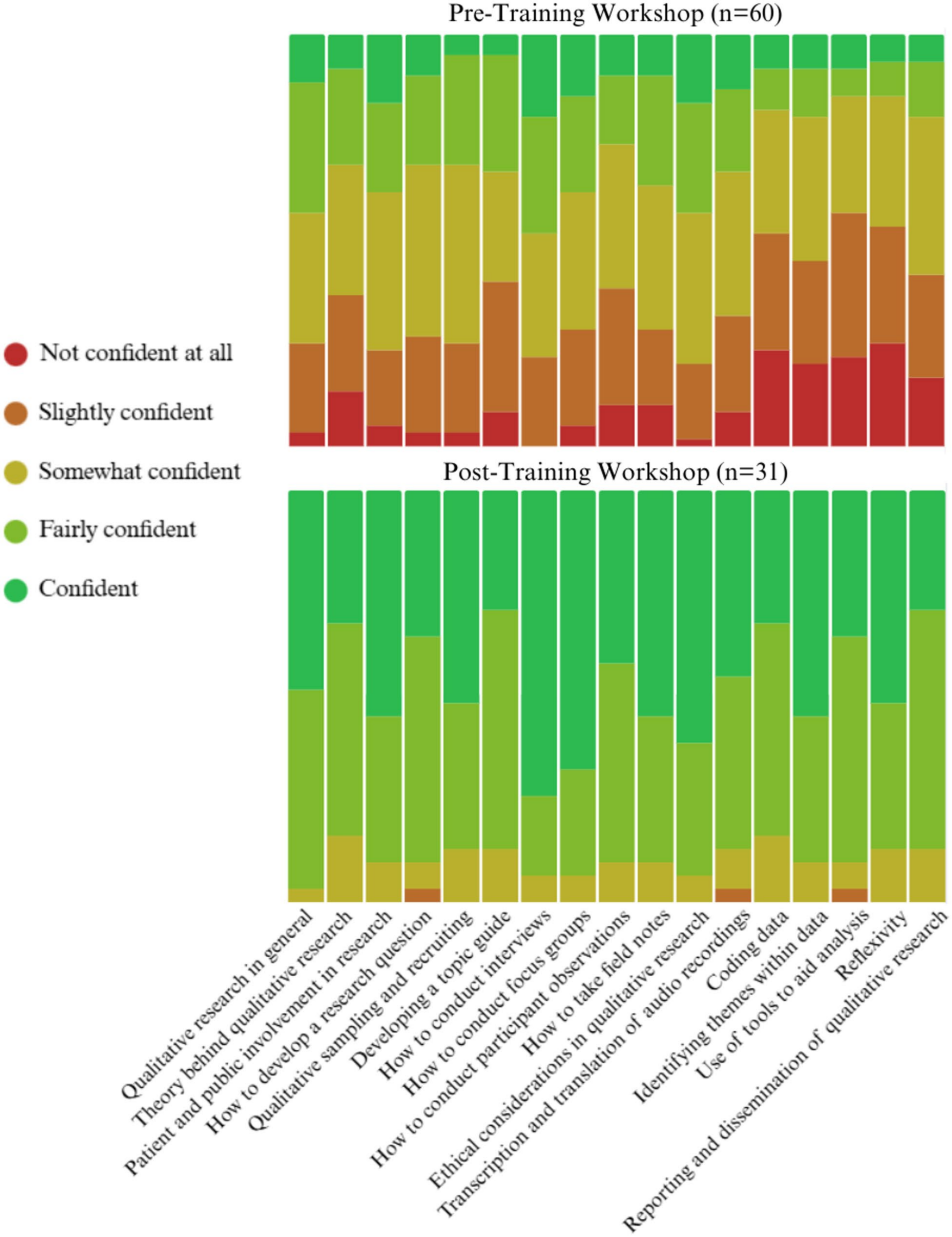
Stacked bar chart of responses to the pre- and post-questionnaire for knowledge and skills

Within the pre-training questionnaire, attendees were asked to rank their experience with qualitative research from zero (no experience) to five (highly experienced), the average score was 2.68. The 31 attendees who completed the post-training questionnaire gave an average score of 4.71/5 for overall satisfaction, up from an average of 2.96 in the pre-training questionnaire (Table 2). Significant improvements were found on all elements addressed within the training. This has been highlighted visually in Fig. 2. We acknowledge the attrition in numbers completing feedback between pre- and post-test which reduces confidence in the positive changes seen, however, this feedback was also supplemented by qualitative feedback from all participants through the questionnaire but also informally during the training.

**Table 2:** Results of mann whitney U test for pre- and post-workshop questionnaire for knowledge and skills

	Pre-Workshop Questionnaire (*n* = 60)		Post-Workshop Questionnaire (*n* = 31)		U	*p*
	Mean	SD	Mean	SD		
Qualitative research in general	3.27	1.04	4.45	0.57	335.5	< 0.001
Theory behind qualitative research	2.9	1.16	4.16	0.69	359.5	< 0.001
Patient and public involvement in research	3.27	1.1	4.45	0.68	370	< 0.001
How to develop a research question	3.12	0.99	4.23	0.72	359.5	< 0.001
Qualitative sampling and recruiting	3.08	0.91	4.39	0.72	273	< 0.001
Developing a topic guide	2.9	1.07	4.16	0.64	332.5	< 0.001
How to conduct interviews	3.47	1.05	4.68	0.6	337	< 0.001
How to conduct focus groups	3.2	1.12	4.61	0.62	288.5	< 0.001
How to conduct participant observations	2.88	1.12	4.32	0.65	283.5	< 0.001
How to take field notes	3.08	1.12	4.45	0.68	302.5	< 0.001
Ethical considerations in qualitative research	3.38	1.03	4.55	0.62	349	< 0.001
Transcription and translation of audio recordings	3.07	1.15	4.29	0.78	377.5	< 0.001
Coding data	2.52	1.20	4.03	0.84	306.5	< 0.001
Identifying themes within data	2.63	1.18	4.1	0.94	333.5	< 0.001
Use of tools to aid analysis	2.45	1.16	3.94	0.85	303	< 0.001
Reflexivity	2.43	1.16	4.06	0.89	272	< 0.001
Reporting and dissemination of qualitative research	2.68	1.11	4.26	0.77	257	< 0.001
Average	2.96	0.93	4.3	0.55	222.5	< 0.001

This is reflected in the feedback from attendees; when asked to provide feedback on their main “takeaway” points, likes and dislikes, and any suggestions to improve future workshops. A range of feedback was reported, including a greater understanding of the benefits of qualitative research.

*“Limitations of qualitative studies are not in themselves a challenge to the authenticity of the results from such studies.” – Attendee 2 (Ghana)*

Attendees also reported increased confidence with certain aspects of qualitative research, such as interviewing technique and analysis.

*“I have a better understanding of various qualitative research techniques*,* including interviews*,* focus groups*,* and observations. I can now more effectively plan and execute qualitative research projects*,* considering factors like sampling*,* data collection methods*,* and analysis techniques. I have learned how to analyse qualitative data systematically and extract meaningful insights. Most importantly*,* the training has boosted my confidence in my ability to conduct high-quality qualitative research.” – Attendee 26 (Ethiopia)*

When asked what they liked most about the workshops, feedback primarily focussed on the appreciation of the practical aspect of the sessions:

*“The training was highly engaging with the audience. As a participant I knew there were questions to respond to at some point. That helped to keep me highly engaged.” – Attendee 27 (Tanzania)*

*“The delivery method was great*,* very interactive and the guided practical sessions gave us an opportunity to retain course content well.” – Attendee 19 (Kenya)*

Regarding what attendees disliked about the course or what they felt could be improved, many reported the brevity of the course, asking for additional time (three or more days) to better pace and delve into certain topics in more depth. Having completed the training, many attendees are looking forward to continuing this community of practice into the future with additional refresher sessions and continued communication with the facilitators.

*“After the training session*,* a [team forum] has been created… to keep the network going and address matters arising with respect to the Qualitative research. Looking forward to a refresher training session.” – Attendee 5 (Ghana)*

*“More training programmes should be organised in the future.” – Attendee 14 (Ghana)*

### Facilitator reflections

The facilitators reflected on what went well during the training.

*“Attendees really enjoyed the engaging activities and ‘real life’ data we presented… We were keen to ensure the training was practical and could facilitate attendees to think about how to apply their learning to their own data.” – Facilitator 1*

During the planning stage, hybrid training was decided upon to balance the pros and cons of both face-to-face and virtual training. This training highlighted the advantages of face-to-face workshops with the increased interactivity of learning for attendees.

*“The in-person attendees were easier to engage with and guide through activities. However*,* multiple efforts were made to optimise the experience of online attendees including a dedicated online facilitator enabling open questions throughout the sessions and the use of breakout rooms.” – Facilitator 7*

*“It felt like a real opportunity to be responsive to the needs of individuals and learn at an appropriate pace… There’s definitely an important role for online methods*,* I hope there’s still a place for [in-person] courses.” – Facilitator 8*

While additional funding to increase the number of days would have been beneficial, the continued development of attendees will require continued support to overcome challenges such as high staff turnover, funding, and mentorship.

*“Supporting qualitative African researchers is a valuable investment that requires multi-layered resources beyond capacity building. We must consider access to funding*,* networking*,* qualitative tools*,* and continuing mentorship.” – Facilitator 5*

*“The next stage beyond this workshop would be to have sustainable ways of increasing qualitative research capacity in SSA.” – Facilitator 3*

It will be important to continue mentorship to ensure a lasting impact from the training.

*“As many of the trainees are in early career stages*,* one can envisage that they will transfer these skills into their future careers… Promoting a community of practice would be one way to foster a lasting impact of the program on public health practice in Africa.” – Facilitator 4*

*“Enhanced confidence and competence in qualitative research methods and application to health policy and practice [we have] strengthened research capacity to address region-specific health challenges.” – Facilitator 6*

## Discussion

This case study provides insights into the possibilities of developing and delivering qualitative training to researchers, and healthcare professionals active in research, in the context of SSA countries. We have demonstrated immediate measures of success and learnings, however, measuring long-term success and impact of the training will only be possible over the next few years.

Materials were developed drawing on successful qualitative research methods teaching at Newcastle University, with tailoring to the context and drawing on specific examples from SSA. The training was developed to be ‘hands on’ and emphasised ‘learning by doing’. Therefore, attendees benefitted through experiential learning and tackling real-world examples. We drew on Kolb’s Experiential Learning Theory to create a transformation experience through experience, reflection, abstract conceptualisation, and active experimentation [23].

In-person attendance was particularly beneficial, allowing for deeper engagement, activities and shared experience. The benefits of in-person teaching have previously been reported, with students citing more effective learning, face-to-face interactions, socialisation and opportunities for collective learning [24]. This was noted by facilitators and attendees who valued the in-person experience. However, since the Covid-19 pandemic, there has been a shift to virtual learning [25]. Virtual or online learning offers a way of dismantling geographical barriers to teaching and learning – a particularly important point when working across multiple countries – though challenges with ‘Zoom fatigue’ and learners disconnecting or not engaging has also been noted [24]. Despite this, even virtual learners felt they had an engaging experience, largely because of the online facilitator who ensured they were involved in every stage, including activities.

As this case study has taken place within the context of a NIHR Global Health Research Group, we hope to build on baseline knowledge to measure the long-term success of the community of practice, including, for example, publications by attendees (both first author and co-author) in high impact qualitative journals and future funding applications involving qualitative work. Presently, not enough time has passed since the training to see the longer-term impact of these workshops on the wider public health workforce. However, we know that repeating the learnings of a topic ensures a deeper and more engaging interaction [26]. To maximise attendee ability to collect, analyse, and publish qualitative data, we have established, and hope to maintain, a community of practice, whereby attendees are encouraged and supported to apply their learnings in public health contexts. With this work being conducted within the wider TraPCAf team, we will continue training and mentoring to ensure a greater impact. Specifically, reviewing the pre- and post-workshop questionnaires, areas of weakness include analysis, specifically coding, identifying themes, and using tools to aid analysis.

Throughout the TraPCAf project, researchers from SSA have been encouraged to take ownership of the interviews they conduct and are being supported with the analysis and dissemination of these data. It is common for researchers in the Global South to lack ownership and continuity with the research they conduct [27]. We aim to ensure this is not the case and that learning can be applied. Ownership and involvement in the qualitative research process will be promoted through a three-component process:

- Regular and continual mentorship by senior qualitative researchers has been maintained through a team forum, attendees have been encouraged to direct any questions and concerns to senior researchers for support.
- Monthly qualitative meetings have been held with the facilitators and attendees, offering an informal space to discuss experiences with data collection, analysis, and dissemination.
- Attendees have been encouraged to take the lead on publication of qualitative research to ensure epistemological justice and the credibility of conclusions drawn from the data with support from senior researchers.

It would be remiss to avoid discussion of the costs relating to qualitative research. Costs involve, for example, salaries, transcription, and software for analysis, and may act as a barrier to conducting qualitative research in the future [17]. As part of the awarded grant, five perpetual qualitative software licenses were purchased for use by attendees and their teams involved in qualitative research as part of TraPCAf. Virtual training was delivered on use of NVivo software, and recorded for longevity, to further enable a growing community of practice where access to software may encourage continued engagement with qualitative research.

## Conclusion

We aimed to upskill qualitative researchers in five countries in SSA to maximise the quality of work on the existing TraPCAf project and empower attendees to carry out independent qualitative research after TraPCAf. To this end, two-day workshops were conducted in Ghana and Kenya with virtual attendees from Nigeria, Ethiopia, and Tanzania, with 58 attendees in total. Recordings of these workshops were then circulated to additional TraPCAf countries for their benefit. The workshops were received positively with improvements in all aspects of knowledge and understanding, including ethical considerations, data collection, and analysis, with qualitative feedback citing a good structure and delivery. To continue these learnings forward, a community of practice has been formed to ensure the long-term impact of this training, with the ultimate goal of improving the quality of qualitative research in these countries. This is enacted through continual engagement and mentorship of workshop attendees, further virtual training and addressing financial barriers to success by funding software access for individuals.

## Supplementary Information

Supplementary Material 1.

Supplementary Material 2.

## Data Availability

The data collected and analysed within this study are available upon reasonable request from the corresponding author.

## Declarations

Clinical trial number: Not applicable.
