# Supplementary material for: Building qualitative research capacity in the global health workforce and developing a community of practice in parts of sub-Saharan Africa: a case study
Source: BMC Med Educ. 2026 Apr 24;26:915. doi: 10.1186/s12909-026-09190-y (PMC13235020; doi:10.1186/s12909-026-09190-y)
Supplement: Supplementary file 1 — Supplementary Material 1. [file 12909_2026_9190_MOESM1_ESM.docx]

Supplementary Materials 1. Agenda of the training workshop and topics covered

**Qualitative Research in Practice**

**Day 1: 9am – 5pm**

| **Time** | **Session** |
| --- | --- |
| 0900 – 0930 | Introduction to qualitative research & aims for the course |
| 0930 – 1000 | Why is qualitative research important |
| 1000 – 1045 | Introduction to methodology and methods in qualitative research |
| 1045 – 1115 | Tea/coffee break |
| 1115 – 1315 | Methods: designing and conducting a qualitative study (Part 1)  *Topic guides & interviews* |
| 1315 – 1415 | Lunch break |
| 1415 – 1500 | Methods: designing and conducting a qualitative study (Part 2)  *Ethics, reflexivity and transcription* |
| 1500 – 1530 | Tea/coffee break |
| 1530 – 1630 | Methods: designing and conducting a qualitative study (Part 2) (cont.) |
| 1630 – 1645 | Patient & Public Involvement / Community Engagement & Involvement |
| 1645 – 1700 | Recap & close |

**Day 2: 9am – 5pm**

| **Time** | **Session** |
| --- | --- |
| 0900 – 0915 | Introduction to Day 2 |
| 0915 – 1100 | Data analysis (Part 1)  *What is qualitative data analysis* |
| 1100 – 1130 | Tea/coffee break |
| 1130 – 1300 | Data analysis (Part 2)  *Coding* |
| 1300 – 1400 | Lunch break |
| 1400 – 1500 | Data analysis (Part 3)  *From coding to themes* |
| 1500 – 1530 | Tea/coffee break |
| 1530 – 1615 | Data analysis (Part 4)  *Managing qualitative data* |
| 1615 – 1645 | Disseminating qualitative research |
| 1645 – 1700 | Recap & close |
