# Supplementary material for: Building qualitative research capacity in the global health workforce and developing a community of practice in parts of sub-Saharan Africa: a case study
Source: BMC Med Educ. 2026 Apr 24;26:915. doi: 10.1186/s12909-026-09190-y (PMC13235020; doi:10.1186/s12909-026-09190-y)
Supplement: Supplementary file 2 — Supplementary Material 2. [file 12909_2026_9190_MOESM2_ESM.docx]

Supplementary Materials 2. Pre- and post- training questionnaire

**Pre-questionnaire**

1.Which country are you from?

Ghana Nigeria Kenya Tanzania Ethiopia

2. What is your current job role?

3. What experience do you have of qualitative research? (1 = No experience, 5 = Highly experienced)

1 2 3 4 5

4. What are your hopes for the qualitative training? (Be as specific as possible)

5. What would this training enable you to do in the future?

6. Rate your confidence in understanding the principles behind, and use in practice, of the following:
(1 = Not confident at all, 5 = Confident in understanding and using in practice)

|  | 1 | 2 | 3 | 4 | 5 |
| --- | --- | --- | --- | --- | --- |
| Qualitative research in general |  |  |  |  |  |
| Theory behind qualitative research |  |  |  |  |  |
| Patient and public involvement in research |  |  |  |  |  |
| How to develop a research question |  |  |  |  |  |
| Qualitative sampling and recruiting |  |  |  |  |  |
| Developing a topic guide |  |  |  |  |  |
| How to conduct interviews |  |  |  |  |  |
| How to conduct focus groups |  |  |  |  |  |
| How to conduct participant observations |  |  |  |  |  |
| How to take field notes |  |  |  |  |  |
| Ethical considerations in qualitative research |  |  |  |  |  |
| Transcription and translation of audio recordings |  |  |  |  |  |
| Coding data |  |  |  |  |  |
| Identifying themes within data |  |  |  |  |  |
| Use of tools to aid analysis |  |  |  |  |  |
| Reflexivity |  |  |  |  |  |
| Reporting and dissemination of qualitative research |  |  |  |  |  |

7. Is there anything else you'd like to add?

**Post-questionnaire**

1. Which country are you from?

Ghana Nigeria Kenya Tanzania Ethiopia

2. Overall, how satisfied were you with the training? (1 = Not satisfied at all, 5 = Extremely satisfied)

1 2 3 4 5

3. Do you feel the course addressed the aspects of qualitative research you hoped it would?

Yes Mostly Not really

4. What is your main takeaway from the training?

5. What did you like most about the training?

6. What did you like least about the training?

7. How could the training be improved?

8. Rate your confidence in understanding the principles behind, and use in practice, of the following:
(1 = Not confident at all, 5 = Confident in understanding and using in practice)

|  | 1 | 2 | 3 | 4 | 5 |
| --- | --- | --- | --- | --- | --- |
| Qualitative research in general |  |  |  |  |  |
| Theory behind qualitative research |  |  |  |  |  |
| Patient and public involvement in research |  |  |  |  |  |
| How to develop a research question |  |  |  |  |  |
| Qualitative sampling and recruiting |  |  |  |  |  |
| Developing a topic guide |  |  |  |  |  |
| How to conduct interviews |  |  |  |  |  |
| How to conduct focus groups |  |  |  |  |  |
| How to conduct participant observations |  |  |  |  |  |
| How to take field notes |  |  |  |  |  |
| Ethical considerations in qualitative research |  |  |  |  |  |
| Transcription and translation of audio recordings |  |  |  |  |  |
| Coding data |  |  |  |  |  |
| Identifying themes within data |  |  |  |  |  |
| Use of tools to aid analysis |  |  |  |  |  |
| Reflexivity |  |  |  |  |  |
| Reporting and dissemination of qualitative research |  |  |  |  |  |

9. Is there anything else you'd like to add?
